# Different Types of Clinical Presentations and Stages of Retinoblastoma Among Children

**DOI:** 10.7759/cureus.10672

**Published:** 2020-09-26

**Authors:** Muhammad Moeez Uddin, Umar Farooque, Muhammad Zunair Aziz, Farah Yasmin, Faisal Qureshi, Yousaf Saeed, Saba Asif, Vijaya Chaitanya Bollampally, Abubakar Tauseef

**Affiliations:** 1 Ophthalmology, Prevention of Blindness Trust Charitable Eye Hospital, Karachi, PAK; 2 Neurology, Dow University of Health Sciences, Karachi, PAK; 3 Internal Medicine, Dow University of Health Sciences, Karachi, PAK; 4 Internal Medicine, Dow University of Health Sciences, Dow International Medical College, Karachi, PAK; 5 Internal Medicine, Ross University School of Medicine, Bridgetown, BRB; 6 Medicine, University at Buffalo, Buffalo, USA; 7 Internal Medicine, Nishtar Medical University, Multan, PAK; 8 Neurological Surgery, Capital Medical University, Beijing, CHN; 9 Internal Medicine, Creighton University, Omaha, USA

**Keywords:** retinoblastoma, leukocoria, strabismus, clinical presentations, children, humans, stages of retinoblastoma

## Abstract

Introduction

Retinoblastoma (Rb) is the most common intraocular malignant tumor of childhood. The different modes of Rb presentation comprise proptosis, anterior chamber inflammatory signs, spontaneous hyphema, secondary glaucoma, and strabismus. The primary aim of this study was to investigate the different clinical presentations and stages of Rb that may help in early detection and timely diagnosis to prevent the advancement of the disease and increase rates of survival in children.

Materials and methods

This was a descriptive cross-sectional study conducted between December 2019 and May 2020 over a period of six months at a tertiary care hospital in Karachi, Pakistan. The sample size included 68 eyes of children with lesions of Rb at the time of presentation to the hospital. Brightness scans (B-scans), computed tomography (CT) scans, and magnetic resonance imaging (MRI) were performed. The International Intraocular Retinoblastoma Classification (IIRC) was used to stage each eye. In case of enucleation (if necessary) of the eye, the biopsy was performed to evaluate the histological features of cancer. All statistical analysis was performed using Statistical Package for Social Sciences version 17.0 (IBM Corp., Armonk, New York).

Results

The mean age of the children was 3.21 ± 1.75 years. Leukocoria was the most common clinical presentation observed in more than half (n = 35, 51.47%) of the sample population followed by proptosis reported in nearly two-fifths (n = 25, 36.76%), strabismus and phthisis bulbi observed in equal proportions (n = 3, 4.41%), and hypopyon documented in a minor proportion (n = 2, 2.94%) of patients. Regarding stages of the Rb disease, the most common stages were observed to be stage C and stage E.

Conclusions

This study concludes that the early detection of Rb is possible through a better understanding of presenting features of the disease. It can prevent the progression of the disease to the advanced stages and decrease morbidity and mortality. The early detection of Rb can be made possible through the examination of red reflex on the regular check-ups of children as leukocoria is the most common clinical presentation.

## Introduction

Retinoblastoma (Rb) is the most frequently reported intraocular malignant tumor of childhood and represents approximately 4% of all pediatric malignancies [1]. It has an incidence rate of 1:14,000-1:20,000 live births [2,3]. The yearly crude incidence ‍of Rb is estimated to be 4.0/100,000 and 2.4/100,000 in children aged below 5 and 10 years, respectively [4]. If not recognized in the early stage of the disease, it may result in the loss of an eye and even death. The latest advancements in technology have enabled prompt diagnosis and effective treatment of Rb, increasing the survival rate of up to 95%. Developing countries are still facing many challenges in achieving this survival rate [1,2]. Moreover, this cancer has no racial and gender predilection [3].

In previous reports, the most common presenting complaints of Rb are leukocoria (22.6%-97.9%) followed by strabismus (5.6%-26%), decreased vision, and proptosis. However, the incidence of proptosis is reported to be relatively higher in developing countries [5-8]. In the subcontinent, this trend prevails with one study demonstrating up to 70% of cases involving a proptosis presentation [9,10]. Similarly, more children with Rb present with later stages of the disease, and studies report International Intraocular Retinoblastoma Classification (IIRC) stages with prevalence ranging as follows: group A (7.5%-62.5%), group B (23.8%-15.6%), group C (6.3%-12.5%), group D (9.4%-38.8%), and group E (23.8%-68.6%) [3,6]. The primary objective of this study was to determine the frequency of different types of Rb clinical presentations among children and stages of their disease. A secondary aim was to determine the impact of sociodemographic factors on the prevalence of different clinical presentations.

## Materials and methods

Study setting and design

A descriptive cross-sectional study was conducted between December 2019 and May 2020 over a period of six months at a tertiary care hospital in Karachi, Pakistan.

Sample size, inclusion, and exclusion criteria

A sample size of 68 eyes was calculated using the World Health Organization (WHO) calculator at an anticipated leukocoria frequency of 22.6% at a confidence interval of 95% and a 5% margin of error [3]. All patients with lesions of Rb at the time of presentation of either gender (i.e., male or female) aged till 10 years whose parents provided written informed consent were included in this study. All patients already receiving treatment for Rb elsewhere and referred to our hospital to treat complications, patients whose parents have not given written informed consent, and those patients who were clinically suspected but not proven to have Rb on histopathology were excluded from this study.

Sampling technique and data collection

A non-probability consecutive sampling technique was employed to collect data. The patients were included in the study after informed consent from the parents (or guardian). After initial diagnosis in the out-patient department (OPD) by a consultant ophthalmologist and admission into an eye ward, a predefined proforma was filled by the investigator regarding the history of the disease. Investigations including brightness scans (B-scans), computed tomography (CT) scans), and magnetic resonance imaging (MRI) were performed. Fundus examination under anesthesia using indirect ophthalmoscope with indentation by the consultant was carried out, and findings were verified. Each eye was staged according to the IIRC criteria. After enucleation (if necessary) of the eye, the biopsy was examined to evaluate the histological features of cancer. Selection bias was minimized by including all the patients in inclusion criteria. Measurement techniques were standardized to further reduce bias.

Statistical analysis

Data were entered and analyzed using Statistical Package for Social Sciences version 17.0 SPSS version 17 (IBM Corp., Armonk, New York). Frequencies and percentages were calculated for categorical variables such as gender, clinical presentations, treatment, family history, and stages of Rb. All continuous variables such as age and duration of the disease were presented as mean and standard deviation. Effect modifiers like age, gender, disease duration, family history, and treatment were addressed through stratification, and the Chi-square test was applied post-stratification. A p value of ≤ 0.05 was taken as statistically significant.

## Results

**Figure 1:**
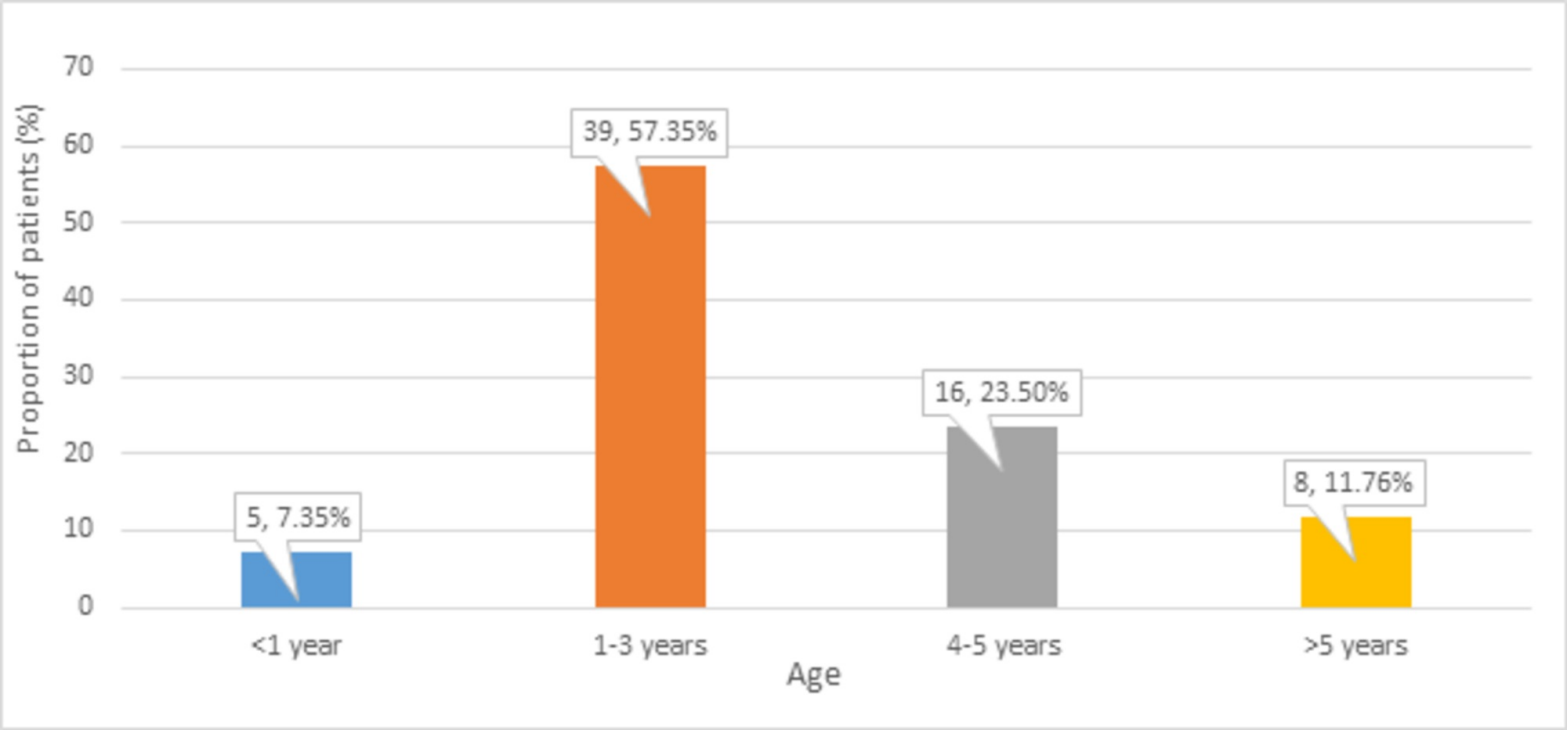
Distribution of participants according to age

The mean age of the children was 3.21 ± 1.75 years. Most of the children (n = 39, 57.35%) were aged one to three years, as shown in Figure *1*.

**Figure 2:**
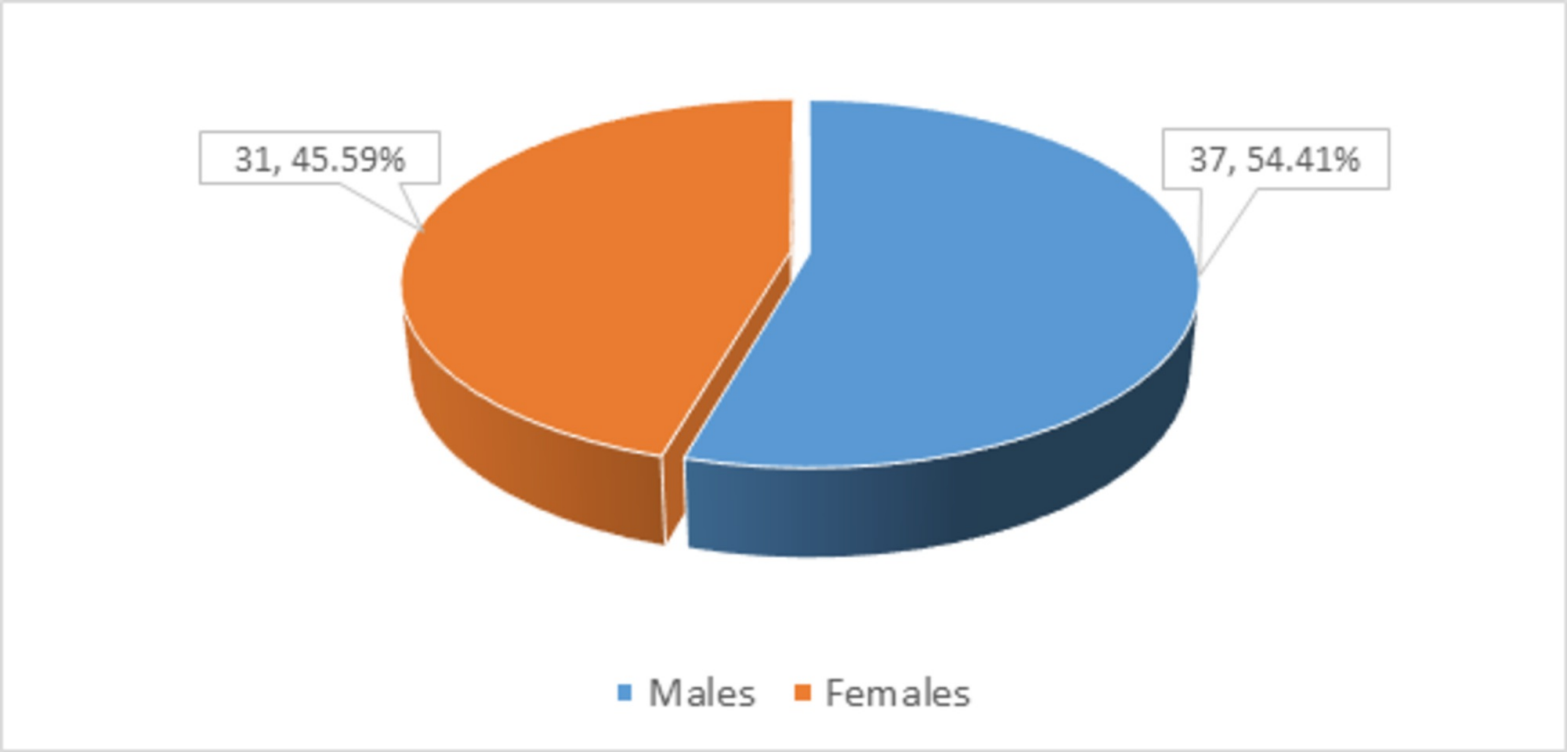
Distribution of participants according to gender

The mean duration of the disease was 4.97 ± 7.29 months. More than two-quarters (n = 37, 54.41%) of patients were males, as shown in Figure* 2*.

**Figure 3:**
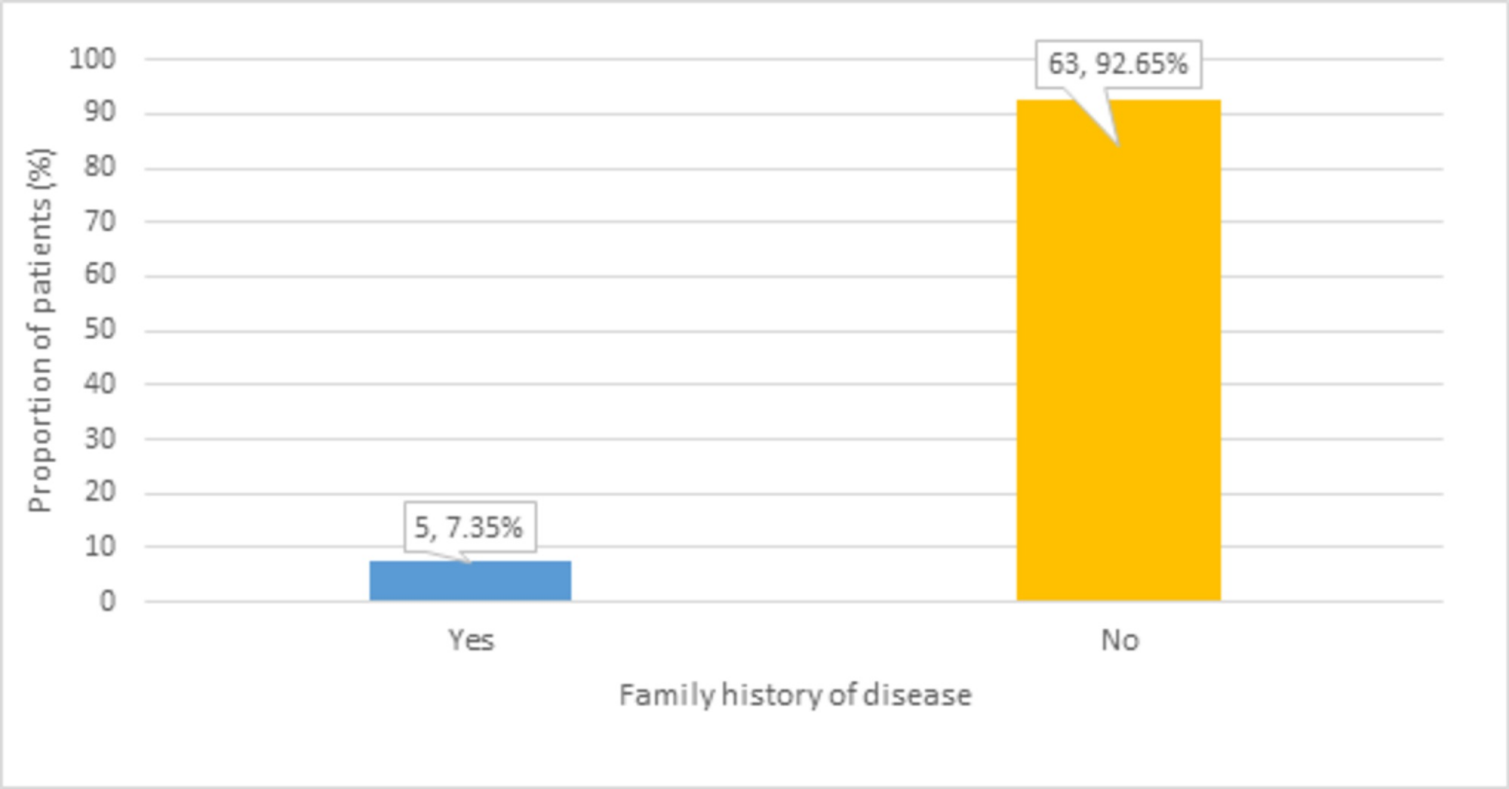
Distribution of participants according to the family history of the disease

Out of 68 patients, a minor proportion (n = 5, 7.35%) had a family history of the disease, as shown in Figure* 3*.

**Figure 4:**
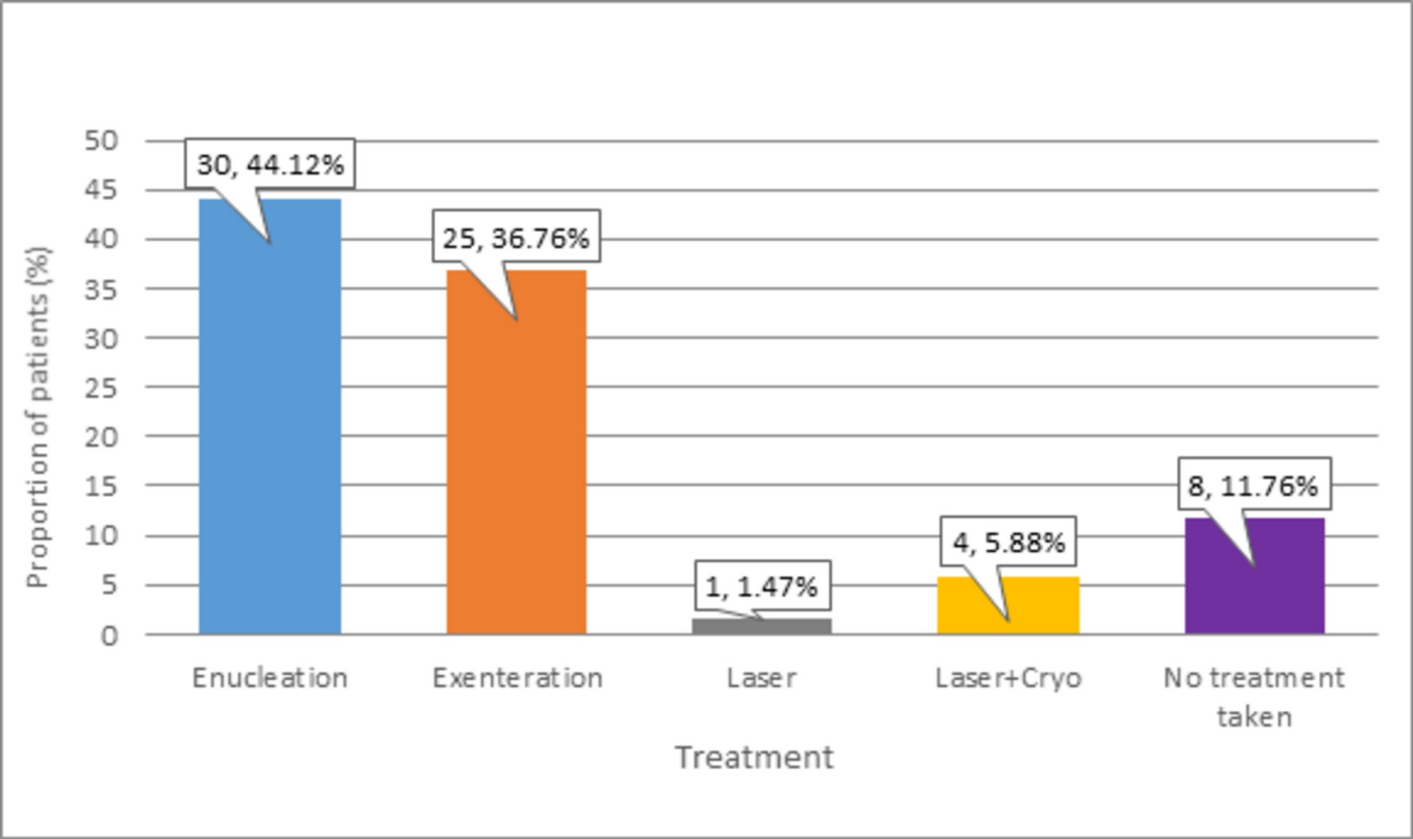
Distribution of participants according to Rb treatment Rb: Retinoblastoma

Enucleation (n = 30, 44.12%) and exenteration (n = 25, 36.76%) treatments were commonly used by our patients, as shown in Figure *4*.

**Figure 5:**
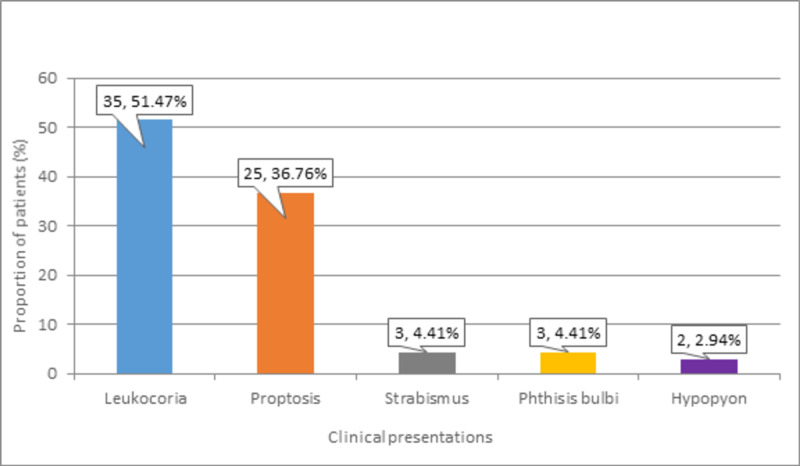
Distribution of participants according to Rb clinical presentations Rb: Retinoblastoma

The most common clinical presentation observed in more than half (n = 35, 51.47%) of patients was leukocoria. Proptosis was reported in nearly two-fifths (n = 25, 36.76%) of patients. Strabismus and phthisis bulbi were documented in equal proportions (n = 3, 4.41%), and hypopyon was observed in a minor proportion (n = 2, 2.94%) of patients. These frequencies of different types of clinical presentations of Rb patients are presented in Figure *5*.

The most common stages of Rb disease were stage C (n = 18, 26.5%) and stage E (n = 30, 44.1%). More than one-third (n = 12, 34.3%) of patients with leukocoria presented with stage C of the disease, while the majority (n = 22, 88.0%) of those with proptosis presented with stage E of Rb disease. Lastly, equal proportions (n = 1, 50.0%) of patients with hypopyon presented with stage C and stage E of the disease. These frequencies of different stages of Rb disease are presented in Table 1.

**Table 1:** Frequency of different stages of Rb disease Rb: Retinoblastoma

Staging	Clinical presentations					Total
	Leukocoria	Strabismus	Proptosis	Phthisis bulbi	Hypopyon	
A	2 (5.7%)	-	-	1 (33.3%)	-	3 (4.4%)
B	6 (17.1%)	-	-	-	-	6 (8.8%)
C	12 (34.3%)	2 (66.7%)	3 (12.0%)	-	1 (50.0%)	18 (26.5%)
D	10 (28.6%)	1 (33.3%)	-	-	-	11 (16.2%)
E	5 (14.3%)	-	22 (88.0%)	2 (66.7%)	1 (50.0%)	30 (44.1%)

Stratification analysis was performed, and the rate of proptosis was significantly higher in patients above three years of age (n = 13, 52.0%) compared to those aged less than or equal to three years (p = 0.037). Other clinical presentations of Rb were not significantly associated with age*. *Rate of leukocoria was significantly associated with the type of Rb treatment as nearly three-quarters (n = 26, 74.4%) of patients with leukocoria received enucleation, while a very minor proportion (n = 2, 5.7%) received exenteration (p = 0.0005). Similarly, the rates of proptosis (p = 0.0005) and phthisis bulbi (p = 0.004) were also significantly associated with type of Rb treatment. Finally, the rate of phthisis bulbi was observed to be significantly associated with the duration of the disease as nearly two-thirds (n = 2, 66.7%) of patients with phthisis bulbi reported a disease duration of > 12 months (p = 0.032). These findings are shown in Table 2.

**Table 2:** Association of Rb clinical presentations with sociodemographic and clinical factors Rb: Retinoblastoma

Variables		Leukocoria n = 35 (51.47%)	Proptosis n = 25 (36.76%)	Strabismus n =3 (4.41%)	Phthisis bulbi n = 3 (4.41%)	Hypopyon n = 2 (2.94%)
Age	≤ 3 years	25 (71.4%)	12 (48.0%)	3 (100%)	3 (100%)	1 (50.0%)
	>3 years	10 (28.6%)	13 (52.0%)	0 (0.0%)	0 (0.0%)	1 (50.0%)
	p value	0.232	0.037	0.547	0.547	0.980
Gender	Male	17 (48.6%)	16 (64.0%)	1 (33.3%)	2 (66.7%)	1 (50.0%)
	Female	18 (51.4%)	9 (36.0%)	2 (66.7%)	1 (33.3%)	1 (50.0%)
	p value	0.319	0.226	0.588	0.663	0.890
Family history	Yes	3 (8.6%)	1 (4.0%)	0 (0.0%)	1 (33.3%)	0 (0.0%)
	No	32 (91.4%)	24 (96.0)	3 (100%)	2 (66.7%)	2 (100%)
	p value	0.692	0.645	0.618	0.208	0.419
Treatment	Enucleation	26 (74.4%)	0 (0.0%)	2 (66.7%)	0 (0.0%)	2 (100%)
	Exenteration	2 (5.7%)	23 (92.0%)	0 (0.0%)	0 (0.0%)	0 (0.0%)
	Laser+Cryo	4 (11.4%)	0 (0.0%)	0 (0.0%)	1 (33.3%)	0 (0.0%)
	No treatment	3 (8.6%)	2 (8.0%)	1 (33.3%)	2 (66.7%)	0 (0.0%)
	p value	0.0005	0.0005	0.394	0.004	0.456
Duration of disease	<1 month	8 (22.9%)	4 (16.0%)	0 (0.0%)	0 (0.0%)	0 (0.0%)
	1-12 months	24 (68.8%)	17 (68.0%)	2 (66.7%)	1 (33.3%)	2 (100.0%)
	>12 months	3 (8.57%)	4 (16.0%)	1 (33.3%)	2 (66.7%)	0 (0.0%)
	p value	0.227	0.948	0.523	0.032	0.611

## Discussion

Rb constitutes the most frequently observed childhood primary ocular malignancy [11]. Globally, about one case of Rb is recorded per 15,000-20,000 live births [12]. The morbidities and mortalities of Rb have declined significantly in developed countries due to the application of better diagnostic and treatment modalities such as focal therapies and chemotherapy rather than conventional treatment options like enucleation and external beam radiation. This has resulted in increased globe preservation and patient survival. The survival rate of Rb patients has reached above 87%-99% in developed countries in the last ten years [13]. The underdeveloped countries, on the other hand, are still facing many challenges in the management of Rb. 

The mean age of the children in this study was 3.21 ± 1.75 years (38.49 ± 20.96 months), and the duration of the disease was 4.97 ± 7.29 months. A study by Noorani et al. also showed patients with a mean age of 3.86 years (SD = 2.56) [14]. The minimum age was one year, while the maximum age was 12 years. In a study by Reddy and colleagues, the mean age of patients at presentation was 24.2 months (range 3-84), while 82.8% of the patients were below 36 months old [5]. This could be explained by the fact that the mean age of Rb diagnosis is 18 months, and a major proportion becomes clinically apparent below three years. Leukocoria was the most common clinical presentation that was observed in 51.47% of our patients, followed by proptosis (36.76%), strabismus (4.41%), phthisis bulbi (4.41%), and hypopyon (2.94%). Similar results were also reported in a study, where leukocoria was the most common presenting clinical feature (71.8%), followed by proptosis (32.8%) [5]. Leukocoria has been reported to be a presenting sign in Rb patients by 22.6% to 97.9%, while the prevalence of strabismus was found between 5.6% and 26% in previous literature [7,15,16]. This difference in the prevalence of clinical presentations could be attributed to varying levels of awareness among the public and the proportion of patients screened. Many uncommon clinical signs have also been reported such as in a study by Abramson et al., which reported inflammatory signs, nystagmus, anisocoria, vitreous hemorrhage, heterochromia iridis, and other findings [8].

Comparing developing countries like Nigeria (84.6%), Nepal (44.2%), Thailand (26.7%), India (25.3%), and Pakistan (52.8%) with developed countries, proptosis has been found to be in high frequencies as a presenting sign [6,7,15,17,18]. A staggering difference in percentage is seen in developed countries like the United States (0.5%) and South Korea (1.4%) while being mostly absent in countries like Australia and Singapore [8,19-23]. This very high incidence of proptosis in developing countries could be explained by the lack of education among the parents regarding the occurrence of eye cancer in young children. This results in parents seeking treatment from traditional faith healers rather than opting for proper cancer diagnosis and management, which leads to the advancement of disease and presentation to the hospital at incurable stages. Leukocoria has many causes including tumors, congenital malformations, vascular diseases, inflammatory diseases, or trauma such as retinal detachment. Strabismus is due to the visual loss caused by a tumor or retinal detachment involving the macula and/or optic disc. Large orbital involvement of the tumor can be staged as moderately advanced, causing pain, proptosis, and ecchymosis, etc. [24].

Lack of awareness in developing countries can cause a delay in seeking treatment. Proptosis is a late presentation that the parents should know is a result of cancer. Reliance on traditional ways of healing can be detrimental and aid in the advancement of the disease to later stages. A fundus examination should be done in children who are suspected to have Rb. Also, awareness about the uncommon signs that may also be rarely associated with Rb should be provided, and a general emphasis needs to be placed on in-time treatment.

## Conclusions

This study concludes that leukocoria is the most common clinical presentation of Rb, and understanding of presenting features of Rb can lead to early detection and prevention of Rb progression to advanced stages that can lead to better outcomes. Educational programs for the general public and parents of Rb patients and monitoring of red reflex on regular child check-ups can be helpful in early diagnosis.
